# Comparative Evaluation of Effects of *Triphala,* Garlic Extracts, and Chlorhexidine Mouthwashes on Salivary *Streptococcus mutans* Counts and Oral Hygiene Status

**DOI:** 10.5005/jp-journals-10005-1530

**Published:** 2018-08-01

**Authors:** Bharathi Padiyar, Nikhil Marwah, Shweta Gupta, Narendra Padiyar

**Affiliations:** 1 Reader, Department of Pedodontics, Mahatma Gandhi Dental College & Hospital, Jaipur, Rajasthan, India; 2 Professor and Head, Department of Pedodontics, Mahatma Gandhi Dental College & Hospital, Jaipur, Rajasthan, India; 3 Professor and Head, Department of Microbiology, Mahatma Gandhi Dental College & Hospital, Jaipur, Rajasthan, India; 4 Principal, Professor and Head, Department of Prosthodontics, Mahatma Gandhi Dental College & Hospital, Jaipur, Rajasthan, India

**Keywords:** Chlorhexidine, Garlic, Mouthwash, Plaque, *Streptococcus mutans*, *Triphala.*

## Abstract

**Aims and objectives:**

To determine and compare the effect of *triphala,* chlorhexidine gluconate, and garlic extract mouthwash on salivary *Streptococcus mutans* count and the oral hygiene status.

**Materials and methods:**

Sixty children aged 9 to 12 years were randomly allocated into the study groups of triphala mouthwash, chlorhexidine mouthwash, garlic extracts mouth-wash, and distilled water mouthwash. Examination included decayed, missing, and filled teeth (dmft)/decayed, missing, filled surface (dmfs) and DMFT/DMFS, plaque index, and S. *mutans* count on days 1, 15, and 30.

**Results:**

The results were statistically analyzed using Friedman test, Wilcoxon signed rank, repeated-measures analysis of variance (ANOVA), paired t-test, one-way ANOVA, *post hoc* Tukey’s honestly significant different (HSD), Kruskal–Wallis test, and Mann-Whitney test; all calculations were done by MEDCALC software 14.0.0 version.

**Discussion:**

*Streptococcus mutans* count had significant reductions using different mouthwashes at 15 days, but the chlorhexidine group showed significant difference from the remaining three groups. Intergroup comparison for plaque index scores of *triphala,* chlorhexidine, and garlic groups was similar in preventing plaque build-up during the 15 days of mouthwash use.

**Conclusion:**

All the three mouthwashes containing triphala, chlorhexidine gluconate, and garlic were comparably efficient in reducing the salivary S. *mutans* count as well as in limiting plaque score; however, chlorhexidine was the most effective in this aspect. In comparison of the two natural ingredients, Triphala is more effective in its antimicrobial effect.

**How to cite this article:** Padiyar B, Marwah N, Gupta S, Padiyar N. Comparative Evaluation of Effects of *Triphala,* Garlic Extracts, and Chlorhexidine Mouthwashes on Salivary *Streptococcus mutans* Counts and Oral Hygiene Status. Int J Clin Pediatr Dent 2018;11(4):299-306.

## INTRODUCTION

Oral diseases, such as dental caries and periodontal disease are major public health problems worldwide and poor oral health has a profound effect on general health and quality of life. Dental caries is still a major health problem in most industrialized countries, as it affects 60 to 90% of school-aged children and the vast majority of adults.^1^ This ubiquitous disease results from the interaction of specific bacteria and constituents of the diet within plaque (a natural biofilm) formed on tooth surfaces. Clarke^2^ was the first to suggest that caries is due to infection of the teeth, which he stated was “by a hitherto undescribed streptococcus, *S. mutans.”*

*Streptococcus mutans* are gram-positive cocci, non-motile, facultative, anaerobic microorganisms, which can metabolize carbohydrates and are considered to be the principal etiological agent of dental caries.^2^ *Streptococcus mutans* is a key contributor to the formation of cariogenic plaque because this bacterium effectively utilizes dietary sucrose to synthesize large amounts of extracellular polysaccharides adheres tenaciously to glucan-coated surfaces, and is also highly acidogenic and acid-tolerant.^3^

The effect of mechanical oral hygiene techniques on the salivary levels of microorganisms, especially mutans streptococci, is of great interest to dentists focused on preventive care. Tooth brushing with fluoridated toothpaste is considered to be the bed-rock of caries prevention. However, tooth brushing alone is effective in reducing bacterial counts in the mouth, but not dramatically and to overcome this issue, it was decided regarding the incorporation of antimicrobial or other chemotherapeutic agents in dental practice.^4^

Mouth rinsing as a formal practice has its first reference credited to Chinese medicine, about 2700 bc, for the treatment of disease of the gums and is the most cost-effective method of preventing dental caries. Mouth rinsing for the prevention of dental caries in children and adolescents was established as a mass prophylactic method in the 1960s and has shown an average efficacy of caries reduction between 20 and 50%.

Chlorhexidine has been studied extensively for over 20 years, and is the gold standard for chemotherapeutic agent against mutans streptococci and dental caries. But since the focus of research is shifting to more natural products or herbally derived antibiotic products in general and specifically in the prevention of dental caries, this study has been devised to evaluate and compare the efficacy of *triphala,* garlic, and chlorhexidine on the salivary *S. mutans* count and oral hygiene status of children.

## AIMS AND OBJECTIVES

To determine and compare the effect of *triphala,* chlorhexi-dine gluconate, and garlic extract mouthwash on salivary *S. mutans* count and the oral hygiene status.

## MATERIALS AND METHODS

A total of 90 students from a residential ashram located in the campus of Mahatma Gandhi University of Medical Sciences and Technology, Sitapura, Jaipur, in the age group of 9 to 12 years were screened for inclusion in the study. The design of the study was randomized clinical blind study. All the students residing in the ashram in the age group 9 to 12 years were subjected to clinical examination and a sampling frame (n = 90) was prepared of those who were at high risk of dental caries, i.e., with DMFT/dmft ≥4 and fulfilled our inclusion criteria specifi-cally of no oral rinse usage. A total of 60 children were randomly allocated into the study groups by using the table of random numbers.

An ethical committee approval was obtained from the Ethical Committee, Mahatma Gandhi Dental College and Hospital, prior to the onset of the study. Verbal consent from children and signed consent forms from the guardian were obtained after the nature of the study and the possible risks were fully explained.

Group I (n = 15) was given 6% *triphala* mouthwash, 10 mL to be used once a day at night for 15 days.

Group II (n = 15) was given 0.2% chlorhexidine mouthwash, 10 mL to be used once a day at night for 15 days.

Group III (n = 15) was given 2.5% garlic extracts mouthwash, 10 mL to be used once a day at night for 15 days.

Group IV (n = 15): Control group—mouth rinsing with distilled water once a day at night for 15 days.

The group allotment was done in such a manner that neither the subjects were told about the content of mouthwash, nor the supervisor trained to administer the mouthwash.

The mouthwashes were prepared under the guidance of the associate professor of Pharmaceutical Sciences at the Central Research Lab of Mahatma Gandhi Medical College, Jaipur.

*Triphala* mouthwash (6%): A quantity of 60 gm *triphala* churna manufactured by The Himalaya Drug Company, Bengaluru, was dissolved in 1000 mL of double de-ionized water and brought to a boil and filtered. To the filtrate, 2 mL of glycerin as a sweetening agent and 1 mL of Pudin Hara, a commercially available pudina extract, were added as a flavoring agent. The solution was cooled and 50 mL was measured and dispensed in amber-colored bottles. The solution was prepared fresh thrice during the study period.

Chlorhexidine mouthwash (0.2%): A commercially available mouthwash (Clohex, Dr. Reddy’s Labs) was used for the study; 50 mL of the mouthwash was dispensed in amber-colored bottles.

Garlic extract mouthwash (2.5%): A quantity of 25 gm of garlic extract powder (pure allicin) sourced from The Himalaya Drug Company, Bengaluru, was added to 1000 mL of de-ionized water and brought to boil and filtered. To the filtrate, 2 mL of glycerin was added as a sweetening agent and 10 mL of Pudin Hara, a commercially available Pudina extract, was added as a flavoring agent. The solution was cooled and 50 mL was measured and dispensed in amber-colored bottles. The solution was prepared fresh thrice during the study period.

Control group mouthwash: For the control group, a mouthwash was prepared by adding 10 mL of Pudin Hara and 2 mL of glycerin to 1000 mL of de-ionized water and then 50 mL was dispensed in amber-colored bottles. The solution was prepared fresh thrice during the study period.

**Fig. 1:**
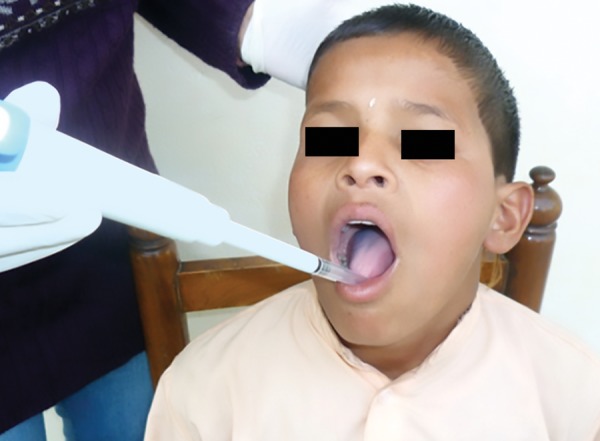
Method of collection of saliva sample

Mitis salivarius bacitracin (MSB) agar media was used for the isolation of *S. mutans.* Each individual’s unstimu-lated whole saliva sample was collected in sterile tubes in the afternoon before lunch with no eating or drinking before lunch for 2 hours, and the subjects were asked to rinse mouth with deionized water for a minute prior to sampling. The pooled saliva was pipetted and collected in a sterile collection tube (Fig. 1). No transport medium was used, as culturing was performed within 30 minutes of collection of samples. The samples were then subjected to microbiological tests. The oral health evaluation of all the children was done using a structured pro forma that consisted of three parts: Demographic details, the history of usage of any antibiotics, corticosteroids, oral hygiene practice, and the third part recorded details of clinical examination pertaining to DMFT and incipient lesions (dmft/dmfs and DMFT/DMFS), plaque index (Turesky Plaque Index ) and *S. mutans* count on days 1, 15, and 30.

**Figs 2A and B:**
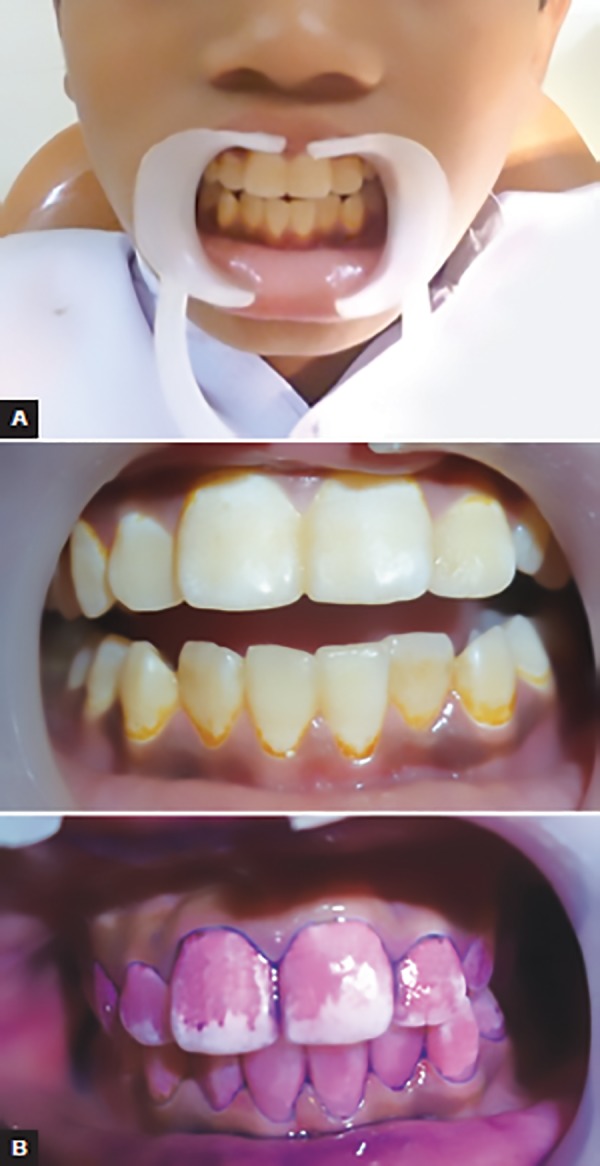
Clinical recording of plaque score

Following collection of baseline data, the subjects were randomly allocated to the four study groups: group I: *triphala;* group II: chlorhexidine; group III: garlic extract; and group IV: control. The children of all the groups were instructed to do regular tooth brushing twice daily. The brushing technique of all the selected children was evaluated and the modified Bass technique of brushing was demonstrated to them repeatedly for a week. It was made sure that brushing technique of all the children was correct, standardized, and effective. The baseline unstimulated saliva was collected from the subjects and inoculated onto MSB agar. The colony counts were obtained by a clinical microbiologist who was blinded to the subject allocation. The existing (baseline) plaque scores of all subjects were recorded by the investigator with the help of a volunteer to record it (Fig. 2). Following this, they received a thorough oral prophylaxis to achieve a zero plaque score. Subjects in each of the groups were given 150 mL of the respective mouthwash, as a daily supervised rinse at night after dinner. The children were advised to take 10 mL of mouthwash in the mouth and swish it in all quadrants of the mouth for a period of 2 minutes. The children were advised not to eat or rinse for the next 30 minutes. They were asked to use the mouthwash for 15 days and compliance was ensured by an adult supervisor of the ashram. Frequent reminders through telephone were given to the supervisor to ensure compliance. At the end of 15 days, unstimulated saliva was collected from the subjects of all the four groups, inoculated onto MSB agar and colony counts were obtained. Plaque index was also recorded at this juncture. For the next 15 days, the subjects in all the groups discontinued the mouthwashes and maintained regular tooth brushing twice a day. After a period of 15 days, the plaque index and the salivary *S. mutans* colony-forming units (CFU) were again evaluated.

### Microbiological Assessment

**Fig. 3:**
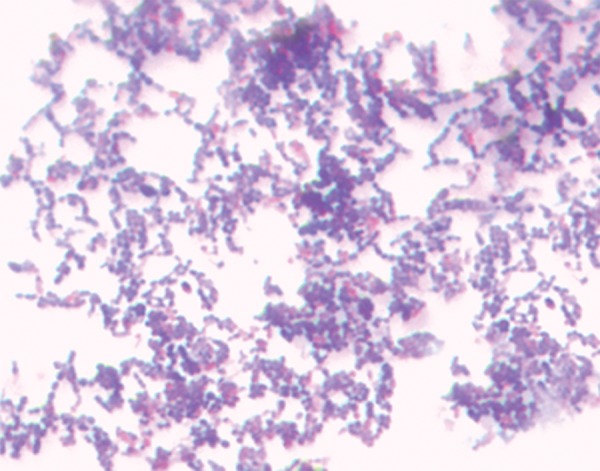
Gram-stained S. *mutans*

Each saliva sample was vortexed vigorously for 30 seconds to ensure a representative mixture throughout the sample prior to the preparation of dilutions and plating. From each sample, 100 μL was taken and diluted in 1 mL of normal saline (10^-1^). The media used in this study for culturing salivary mutans streptococci was MSB agar. A 1μL volume from each of the dilutions was pipetted onto separate agar plates and evenly spread onto the agar surface using sterile spreaders. The plates were then incubated aerobically at 37°C for 48 hours under 5 to 10% CO_2_. To avoid bias, all plates were processed and examined by the same investigator. Colonies observed as round or spherical, raised, convex, black in color, ranging from a pinpoint to pinhead size with a rough surface were identified as *S. mutans* colonies in *triphala,* garlic, chlorhexidine, and control groups at baseline and 15 and 30 days respectively. Identification of *S. mutans* was confirmed by biochemical tests like catalase test and mannitol and sorbitol fermentation (Fig. 3). The colony count of each plate was recorded and the mean CFU/mL was determined after multiplying the colony count of each plate with its respective dilution factor.

## RESULTS

Data were entered in an Excel sheet to prepare the master chart and was transported to statistical software for analysis. Continuous data were summarized as mean and standard deviation, while ordinal data as median. For “within group” comparison, Friedman test and Wilcoxon signed rank test were used to analyze ordinal data, while repeated-measures ANOVA and paired t-test for continuous data. For “between group” comparison, one-way ANOVA and *post hoc* Tukey HSD were used for continuous data, whereas Kruskal–Wallis test and Mann-Whitney test for ordinal data. “p” value <0.05 was taken as significant. All calculations were done by MEDCALC software 14.0.0 version.

**Graph 1:**
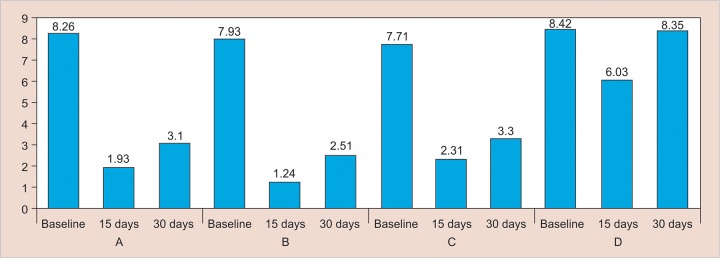
Comparison of mean S. *mutans* count

**Graph 2:**
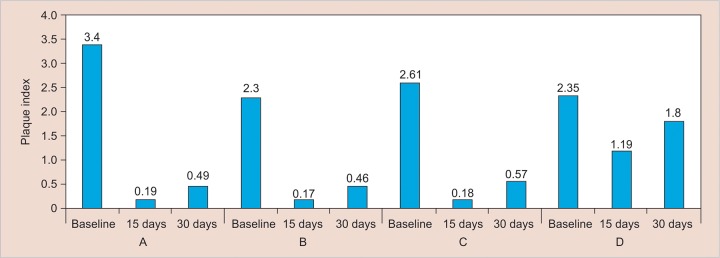
Comparison of median plaque index

Table 1 (Graphs 1 and 2) shows the intergroup comparison of *S. mutans* counts at three different points of observations. At baseline, on applying one-way ANOVA, the mean *S. mutans* counts of all the four groups were found alike. At 15 days, mean *S. mutans* counts of all four groups were found alike (one-way ANOVA). *Post hoc* Tukey HSD test showed that *S. mutans* counts in group I differed significantly from groups II and IV, but not with group III. Group II differed significantly from all the remaining three groups in reducing the *S. mutans* counts, also being the most effective. Group III differed significantly from groups II and IV, but not with group I. Group IV, being the control, differed significantly from the rest of the groups showing the least decrease in *S. mutans* counts. Groups I and III are similar, but significantly less effective than group II. At 30 days, comparison between groups shows the same pattern as found at 15 days.

Table 2 shows the plaque index scores of all the groups at three different points of observations. On applying Kruskal–Wallis test, the plaque index scores of the four groups were not alike at baseline (p = 0.008), 15 days, and 30 days (p < 0.001).

## DISCUSSION

Modern concepts consider caries as an interaction between genetic and environmental factors in which social, behavioral, psychological, and biological factors are expressed in a highly complex and interactive manner. Chemotherapeutic agents have a key role as adjuncts to mechanical methods for preventing and treating peri-odontal disease, and mouthwashes are widely used as an adjunct to mechanical oral hygiene procedures for their analgesic, anti-inflammatory, antimicrobial activity, and anticaries activity.^5^ Several adverse effects have been attributed to the use of mouthwashes currently available in the market, such as taste alteration, unpleasant taste, increased risk of caries due to fermentation and alcohol content, and discoloration of teeth. This has stimulated the research for alternatives that are more appropriate for young children. One approach to overcome this problem is through the use of home-made remedies that are easily obtainable, safe, effective, and acceptable to all.^6^ “The journey from the art of filling teeth to the science of prevention” is exemplified by the recent global trend of going green toward natural resources.

**Table 1:** **Table 1:** Intergroup comparison of S. *mutans* count

										*ANOVA*					
*Time interval*		*Group*		*n*		*Mean*		*Standard deviation*		*F*		*p-value*		Significant difference from*	
Baseline		I		15		8.26 × 10^5^		0.80 × 10^5^		2.083		0.113			
		II		15		7.93 × 10^5^		0.86 × 10^5^							
		III		15		7.71 × 10^5^		0.88 × 10^5^							
		IV		15		8.42 × 10^5^		0.87 × 10^5^							
15 days		I		15		1.93 × 10^5^		0.38 × 10^5^		372.662		<0.001		II, IV	
		II		15		1.24 × 10^5^		0.33 × 10^5^						I, III, IV	
		III		15		2.31 × 10^5^		0.43 × 10^5^						II, IV	
		IV		15		6.03 × 10^5^		0.55 × 10^5^						I, II, III	
30 days		I		15		3.10 × 10^5^		0.42 × 10^5^		331.440		<0.001		II, IV	
		II		15		2.51 × 10^5^		0.51 × 10^5^						I, III, IV	
		III		15		3.30 × 10^5^		0.58 × 10^5^						II, IV	
		IV		15		8.35 × 10^5^		0.75 × 10^5^						I, II, III	

*Tukey HSD

**Table 2:** **Table 2:** Plaque index at different time intervals

										*Kruskal–Wallis test*			
		*Groups*		*n*		*Mean rank*		*Median*		*Chi-square*		*p-value*	
Baseline		I		15		43.23		3.4		11.91		0.008	
		II		15		22.17		2.3					
		III		15		28.13		2.61					
		IV		15		28.47		2.35					
15 days		I		15		24.33		0.19		33.41		<0.001	
		II		15		22.50		0.17					
		III		15		22.17		0.18					
		IV		15		53.00		1.19					
30 days		I		15		23.97		0.49		36.90		<0.001	
		II		15		16.47		0.46					
		III		15		28.57		0.57					
		IV		15		53.00		1.8					

Clinically, plaque formation was measured by using Turesky-Gilmore-Glickman modification of the Quigley Hein plaque index, as this modification is recognized as a reliable index for measuring plaque, using an estimate of the area of the tooth covered by plaque. Plaque was assessed on the labial, buccal, and lingual surfaces of all the teeth after using a disclosing agent. The same plaques coring index was also used in a study by Narayan and Mendon.^7^

A suitable culture media of MSB agar comprising of 90 gm mitis salivarius dehydrated agar (Hi Media); 1 mL 1% potassium tellurite (Hi Media), w/v sucrose (20%); 0.2 U bacitracin (Hi Media); distilled water was used for culturing *S. mutans.* This medium (with 1% potassium tellurite) is a highly selective medium, which enables to isolate streptococci from highly contaminated specimens, as it inhibits a wide variety of bacteria.^8^

The children of age group 9 to 12 years were selected for the study as globally, the weighed mean DMFT among 12-year-old children is 1.67 (2011) and in South East Asian region, the increase in DMFT is from 1.12 (2004) to 1.87 (2011) among 12-year-old children.^9^

*Triphala* is one of the well-known Indian Ayurvedic herbal formulations consisting of dried and powdered fruits of three medicinal plants, namely *Terminalia bellerica, Terminalia chebula,* and *Emblica officinalis. Triphala* can be used for dental diseases as mentioned in sushruthasam-hita, as it showed wound-healing property when applied on wounds externally. Date and Kulkarni^10^ in a study found that *T. chebula* strengthens the gums, prevents and treats several diseases, such as dental caries, bleeding gums, and stomatitis. A study by Jagdish et al^11^ reported a reduction of 83% of *S. mutans* at 5% concentration and 86% reduction at 10% concentration in *in vitro* study. In our study, a comparison between the baseline, 15 days, and 30 days plaque scores was found to be highly significant, suggesting that the mouthwash had good results in short duration of time. This reduction in plaque scores could be attributed to the antibacterial activity of *triphala,* and strong antioxidant effect due to belerica was the most active ingredient, as phenolic nature may be responsible to scavenge the free radicals, thus having the antioxidant effect. The effect of *triphala* mouthwash on plaque index scores also showed a similar pattern over the period of study. The plaque formation had significantly reduced at 15 days. At 30 days, it showed slight increase. The substantivity from baseline to 30 days, the plaque formation was comparatively very much reduced showing that *triphala* was effective in preventing the plaque formation.

Chlorhexidine is a bis-biguanide that was first described by Hassan et al.^5^ It is an antimicrobial agent with special affinity for oral structures and has a long history as a substance for inhibiting plaque formation. Chlorhexidine has been considered as the “gold standard” and is highly effective in reducing the oral microbial load. The *S. mutans* count of group II depicted shows that the efficacy of chlorhexidine was significant from baseline to 15 days time period, when the subject was using the mouthwash. At 30 days, the *S. mutans* count increased when the mouthwash was discontinued. However, *S. mutans* count at 30 days showed significant decrease compared with baseline, demonstrating chlorhexidine substantivity even after discontinuing the mouthwash. The effect of chlorhexidine mouthwash on plaque index scores also showed a similar pattern over the period of study. The plaque formation had significantly reduced at 15 days; at 30 days, it showed slight increase. The substantivity from baseline to 30 days, the plaque formation was comparatively very much reduced, showing that chlorhexidine was effective in preventing the plaque formation. In this study also, chlorhexidine mouthwash demonstrated very good efficacy and substantivity. The results of this study are in accordance with those of Van Strydonck et al^12^ and Persson et al.^13^

Garlic *(Allium sativum)* has always been known to play an important role in the field of medicine throughout the history of mankind and has been shown to have potential inhibiting effect on *S. mutans* harbored in dental plaque.^14^ Chemical analyses of garlic cloves have shown the presence of sulfur-containing compounds like allicin which firstly functions as an antioxidant, secondly, its ability to attach the sulfur (SH) groups in enzymes, proteins, and modify their activities, thereby inhibiting the sulfhydryl enzymes, and thirdly, its ability to rapidly penetrate into cells through the cell membranes. In this study, we found significant change in *S. mutans* count of the garlic mouthwash group. The effect of Garlic mouthwash on plaque index scores also showed a similar pattern over the period of the study in accordance with similar studies by Chavan et al^6^ and Prabhakar et al.^14^

In this study, when the different groups were compared using Tukey HSD *post hoc* test with p < 0.001, the *S. mutans* count in all the groups at baseline did not show any statistically significant difference (p = 0.113). When we compared the efficacy of the different mouthwashes at 15 days, we observed significant reductions in the *S. mutans* count in all the mouthwashes including the control. However, the chlorhexidine group showed significant difference from the remaining three groups, confirming its label as the gold standard as a preventive agent against dental caries. *Triphala* and garlic groups showed statistically significant difference compared with the chlorhexidine group and the control group, but not against each other. Hence, *triphala* and garlic mouthwashes show similar efficacy against *S. mutans,* even though *triphala* showed slightly better results. The control group, as one would expect, was considerably less effective compared with the remaining test groups. The fact that the control mouthwash contained all the common ingredients other than the active ingredient confirms the effectiveness of the test ingredients used in the study. Bajaj and Tandon^15^ conducted a study to ascertain the effects of a mouthwash prepared with *triphala* on dental plaque, gingival inflammation, and microbial growth, and compare it with commercially available chlorhexidine mouthwash. It was concluded that there was no significant difference between the *triphala* and the chlorhexidine mouthwash. Narayan and Mendon^7^ compared plaque formation at 24 hours after the use of *triphala,* Hi Ora, chlorhexidine, and Colgate Plax mouth-washes. They concluded that *triphala* and Hi Ora present an antiplaque efficacy similar to that of chlorhexidine, and were more effective at inhibiting plaque formation than Colgate Plax.

Intergroup comparison using the Kruskal–Wallis test for plaque index scores showed a significant difference in scores between the groups at baseline (p = 0.008). However, it is of no significance to the study, as the plaque scores of all the subjects across the groups were made zero before the mouthwashes were administered. After 15 days, all the groups showed statistically significant differences between each other. At 15 days, comparison between *triphala* and chlorhexidine, between chlorhexi-dine and garlic, and between *triphala* and garlic did not show statistically significant difference, showing that *triphala,* chlorhexidine, and garlic groups were similar in preventing plaque build-up during the 15 days of mouthwash use.

When the different groups were compared using Tukey HSD *post hoc* at 30 days for substantivity, it showed that the chlorhexidine group showed statistically significant difference from the other three groups, reflecting the same results as the efficacy at 15 days. *Triphala* and garlic groups differed significantly from chlorhexidine and control, but not from each other, reflecting that both show comparable substantivity in reduction of *S. mutans* count at 30 days. The control group showed significant difference from the rest of the groups. The substantivity of chlorhexidine was found to be better than *triphala* and garlic mouthwashes. Its effectiveness is attributed to its substantivity (i.e., its ability to maintain therapeutic activity for a prolonged period of time), which is facilitated by its adsorption onto tooth surfaces, pellicle, plaque, and mucous membranes, and cationic nature of chlorhexidine that enables it to bind to the tooth surface pellicle, plaque, and mucous membranes.^16^

## CONCLUSION

All the three mouthwashes containing *triphala,* chlorhexi-dine gluconate, and garlic were comparably efficient in reducing the salivary *S. mutans* count as well as in limiting plaque score; however, chlorhexidine was the most effective in this aspect. In comparison of the two natural ingredients, i.e., *triphala* and garlic, the former is more effective in its antimicrobial effect. Most of the studies that are mentioned in literature either compare the natural components with each other or compare one of the natural additives with chlorhexidine. The uniqueness of this study was that it not only compares two natural ingredients *(triphala* and garlic) with each other and chlorhexidine but also evaluates the substantivity of each active ingredient. However, further long-term clinical studies to assess substantivity are needed to substantiate the therapeutic use and evaluate any potential side effects of *triphala* and garlic mouthwashes. In conclusion, we can say that chlorhexidine mouthwash is the most superior mouthwash in its active and passive action, especially on salivary *S. mutans,* but *triphala* and garlic mouthwash are effective in the active mode and are good options for a natural replacement of mouthwash and their role needs to be extensively evaluated.
